# Mammography using low-frequency electromagnetic fields with deep learning

**DOI:** 10.1038/s41598-023-40494-x

**Published:** 2023-08-15

**Authors:** Hamid Akbari-Chelaresi, Dawood Alsaedi, Seyed Hossein Mirjahanmardi, Mohamed El Badawe, Ali M. Albishi, Vahid Nayyeri, Omar M. Ramahi

**Affiliations:** 1 Department of Electrical and Computer Engineering, University of Waterloo, Waterloo, ON N2L 3G1 Canada; 2 Department of Electrical Engineering, Taif University, 26571 Taif, Saudi Arabia; 3 Department of Medical Physics, Stanford University, Stanford, CA 94304 USA; 4 Soundskrit Inc., 1751 Rue Richardson, No. 5102, Montreal, Canada; 5 Electrical Engineering Department, King Saud University, 11421 Riyadh, Saudi Arabia; 6 School of Advanced Technologies, Iran University of Science and Technology, Tehran, 16846-13114 Iran

**Keywords:** Biomedical engineering, Medical imaging

## Abstract

In this paper, a novel technique for detecting female breast anomalous tissues is presented and validated through numerical simulations. The technique, to a high degree, resembles X-ray mammography; however, instead of using X-rays for obtaining images of the breast, low-frequency electromagnetic fields are leveraged. To capture breast impressions, a metasurface, which can be thought of as analogous to X-rays film, has been employed. To achieve deep and sufficient penetration within the breast tissues, the source of excitation is a simple narrow-band dipole antenna operating at 200 MHz. The metasurface is designed to operate at the same frequency. The detection mechanism is based on comparing the impressions obtained from the breast under examination to the reference case (healthy breasts) using machine learning techniques. Using this system, not only would it be possible to detect tumors (benign or malignant), but one can also determine the location and size of the tumors. Remarkably, deep learning models were found to achieve very high classification accuracy.

## Introduction

The absence of reliable, non-invasive, and high-resolution technologies to detect breast malignancies at early stages has resulted in increased morbidity and poor quality of life for many women. Current technologies have been facing critical challenges that are associated with health-related issues, scanning time, and affordability. Considering the fact that breast cancer is the most common cancer amongst women, proposing new techniques for breast cancer detection, which can eliminate the aforementioned challenges and improve the diagnosis resolution, is of significant importance^1^. Such techniques can lead to early-stage detection, thereby increasing the chance of full recovery^2^.

One of the traditional methods for cancer detection is X-ray mammography. Although this technology is relatively inexpensive and suitable for detecting malignant tissues in low-density breasts, it has a potentially harmful effect due to ionizing radiation. For high-density breasts, diagnosis of cancerous tumors is challenging owing to a high overlap between fat and malignant tissues^3,4^. Magnetic resonance imaging (MRI) is used as a complementary diagnosing tool, providing higher accuracy than X-ray mammography, especially for high-density breasts. However, being costly, MRI cannot be used for regular screening, particularly in low-income communities^5^. Ultrasound is also used for breast-cancer diagnoses; however, its accuracy in detecting tumors depends on the radiologist’s expertise^6^.

In recent years, breast cancer detection techniques based on microwave imaging (MWI) have been introduced as an alternative to the aforementioned traditional methods^7–12^. MWI uses non-ionizing electromagnetic (EM) waves instead of potentially hazardous ionizing waves. In addition, low-frequency electromagnetic excitation allows for deeper penetration, thereby increasing the ability to diagnose anomalies buried deep inside denser breasts^13,14^. Furthermore, MWI takes advantage of low-cost system integration. The main components of any MWI-based system are transmitters (antennas) to emit EM waves and receivers (detectors) to capture the data in the form of the EM field distribution, which is correlated to the permittivity of tissues^15,16^. The collected data is then sent to a computer for image construction.

Generally, technologies based on MWI can be categorized as radar-based imaging technology and microwave tomography^17^. The radar-based imaging technique constructs images using reflected waves from the objects under examination^18^, whereas microwave tomography techniques are mainly founded on inverse scattering algorithms to construct an image of the object^19^. Nevertheless, all current technologies based on MWI have been facing critical challenges such as the complexity of the system (due to using multiple antennas), the need for impedance-matching liquid between the breast and antennas in a wide variety of cases, the complication of the image construction using experimental data, and low accuracy and resolution owing to the coupling between the antennas (detectors)^10,13^. Microwave-based systems have also been introduced for breast cancer detection without the need for full breast imaging^20,21^.

In a recent work, we introduced a new microwaves-based modality for breast cancer detection, which is identical in concept to X-ray mammography but using microwaves^22^. The key concept behind this new and simple modality is achieving an impression of the female breast that correlates to the constituents of the breast. This impression is captured using a metasurface consisting of an ensemble of electrically-small resonators stitched to achieve good electromagnetic energy absorbance. Then, artificial intelligence is used to train the system to provide a conclusion as to the probability of the existence of a tumor within the breast. The developed system in^22^ has two fundamental limitations. The first is due to the metasurface used to capture the impressions. This metasurface is constrained by its unitcell size, which directly relates to the frequency at which the system can operate. To achieve higher impression resolution, the metasurface needs to have a higher number of smaller cells (which resemble pixels). However, smaller cells typically resonate at higher frequencies, limiting the penetration of the electromagnetic field into the breast. The second challenge relates to the non-highly accurate impressions achieved. Since a relatively high frequency was used in^22^ (around 2.0 GHz), the achieved impressions could not give a robust correlation to all the constituents of the breast, again, for the simple reason that at 2.0 GHz, the penetration into the breast is shallow and does not cover the entire volume of the breast^23^.

Deep neural networks have shown remarkable performances across various medical imaging domains, performing tasks such as classification, detection, segmentation, and reconstruction. Artificial neural networks (ANNs) require a large amount of high-quality data for efficient training and to achieve reliable outputs. Hence, part of their successes is indebted to the abundant data collected from widely used imaging modalities, including MRI, computerized tomography (CT), ultrasound, and pathology. While the implementation of ANNs in the microwave domain dates to more than two decades ago by training shallow networks (containing one fully connected hidden layer)^24^, only recently attempts were reported to use deep networks^25,26^. One of the underlying reasons is due to data scarcity of microwave images. The recent works proposed a conventional convolutional neural network (CNN) to enhance the microwave imaging resolution, while the earlier works used an auto-encoder followed by a secondary CNN for image reconstruction purposes.

Deep learning (DL) algorithms are conventionally categorized into supervised learning, weakly supervised learning, and unsupervised learning. In supervised learning, a set of *M* training samples $\{(x_i,y_i)\}^{M}_{i=1}$ exist where for each input $x_i$ an annotated label, $y_i$ is available. The objective is to train the network $g_{\theta }: x \rightarrow y$ based on minimizing a loss function $\mathcal {L}$ that predicts the most accurate label for an unknown test image. Weakly supervised methods leverage only the coarse-grained annotations to deduce the fine-grained labels, while unsupervised learning requires no annotations and aims at recognizing patterns in the data.

Here, our main objective is to provide the majority of women who are under risk of breast cancer worldwide with a modality that can be used frequently. As discussed earlier, MRI modality is neither affordable nor convenient for frequent screening. Also, x-ray mammography can cause health issues for patients due to potentially ionizing radiation. Our proposed modality is most suitable for screening purposes, owing to its non-hazardous, low-cost, and easy-to-use features.

In this work, we present a design of a mammography system for detecting tumors using low-frequency electromagnetic fields (LFEMF) that overcomes the challenges in our earlier work^22^. First, instead of using a plane wave excitation that requires a horn antenna placed in the far field of the breast under examination, a simple electrically-small dipole antenna is used in the near field of the breast to provide a rich source of excitation that includes EM energy, containing all field polarizations. This type of excitation is intended to excite all constituents of the breast, thereby creating an impression that is inclusive of most breast tissues. Second, to enable a much lower frequency excitation and, as a result, effectively increase the penetration and impression resolution, we have designed a metasurface made up of an ensemble of highly miniaturized unitcells that operate at 200 MHz (which is one order of magnitude reduction as compared to the earlier design^22^). Third, we employ a new technique in the image processing stage, which is based on the comparison between healthy and unhealthy breast models. In fact, after capturing the images, unlike the previous work^22^, the dataset images will first be pre-processed and then fed into the neural network algorithm to train the introduced architecture in a supervised manner.

## Unitcell design

The metasurface is analogous to the X-ray film used in X-ray mammography. It is effectively an ensemble of electrically-small resonators, which are typically referred to as unitcells. When the unitcells are placed in close proximity to each other, they will create a tightly-spaced array, whereby the input impedance of each unitcell is strongly altered by the surrounding ones. The unitcell can be designed using different topologies available in the literature, one of which is electric-inductive-capacitive (ELC) resonators that provide high sensitivity to incident electric fields^22,27^. Such a unitcell is made of capacitors and inductors, realized by gaps and conductive ring patterns. An important design feature of the metasurface for our application is being low loss to maximize energy absorption at the terminals. The earlier works have shown that a metasurface composed of ELC resonators can achieve a near-unity absorption, however, for energy harvesting applications.

Since our desired frequency is around 200 MHz ($\lambda$ = 1.5 m), the typical size of an ELC resonator would be large compared to the human female breast. This would then preclude the possibility of using the unitcell as a pixel for constructing an impression of the breast. Essentially, the resolution of the impression would be directly dependent on the size of the unitcell. To enable sufficient miniaturization of the unitcell such that the metasurface would have a reasonable number of them for high resolution, we can increase the equivalent capacitance and/or inductance, thereby decreasing the frequency according to Eq. (1).

1 $$\begin{aligned} f_r = \frac{1}{2\pi \sqrt{LC}}, \end{aligned}$$

where $f_r$ is the resonance frequency, and *C* and *L* are the equivalent capacitance and inductance of the ELC resonator, respectively. To increase the capacitance, the separation between the metallic parts of the resonator will have to decrease significantly. While this is possible from a fabrication standpoint, it would, however, escalate the fabrication tolerance and cost. Therefore, our approach is to increase the inductance using lumped inductors placed in the inner and outer rings of the proposed ELC resonator. The concept of manipulating the resonance frequencies by lumped elements, such as inductors, capacitors, and resistors, for miniaturization of electrically-small resonators, has been made use of in previous works such as^28^ and references therein. Later, the resonance frequency can be tuned to optimize the S-parameters for maximum absorption.

**Figure 1:**
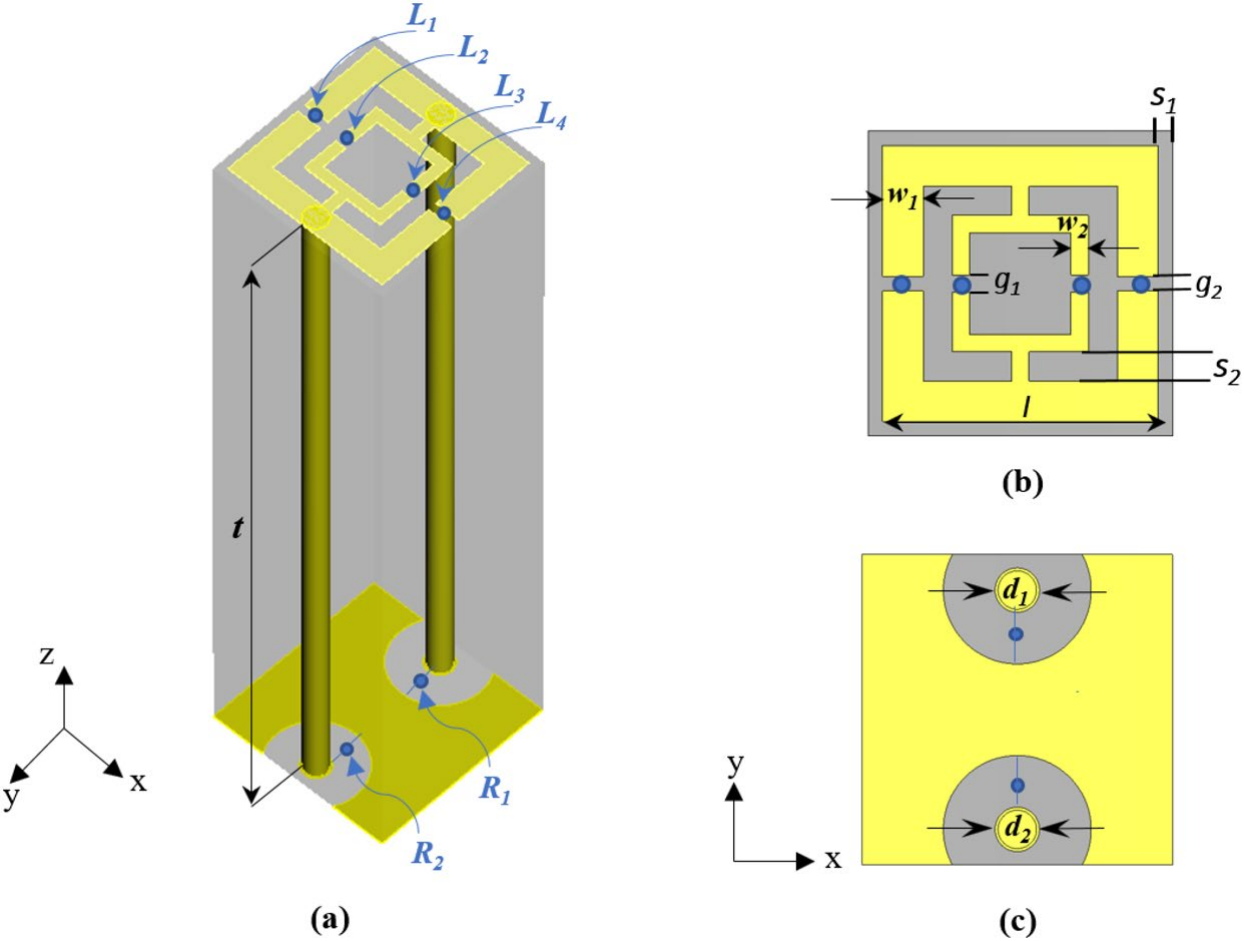
(**a**) 3D view of the unitcell, (**b**) top view, and (**c**) bottom view.

**Table 1:** Design parameters of the unitcell.

Parameter	Value	Parameter	Value
$L_{1,2,3,4}$	100 nH	$R_{1,2}$	470 $\Omega$
$w_1$	1.4 mm	$w_2$	0.6 mm
$g_{1,2}$	1 mm	*l*	9.5 mm
$s_1$	0.5 mm	$s_2$	1 mm
$d_{1,2}$	1.3 mm	*t*	4 cm

The proposed unitcell, which is a miniaturized ELC resonator, is shown in Fig. 1. So as to preserve the unitcell symmetry in the x and y directions, not only have the splits been selected at the middle of the rings, but also the values of the inductors and resistors were selected to be identical. In addition, two vias, instead of a single via, were implemented to retain a degree of symmetry vis-a-vis the impinging electromagnetic field^29^. To minimize the loss, in our design, we considered a low-loss Rogers substrate (TMM10i) with a relative permittivity of $\epsilon _r$ = 9.8 and a loss tangent of tan($\delta$) = 0.002.

The unitcell was modeled using CST Microwave Studio^30^. Considering that the unitcell is intended to be placed in an infinitely self-repeating structure in both x and y directions, in order to model the structure periodicity, a perfect magnetic conductor boundary condition (PMC boundary condition) was placed in the x-direction, and a perfect electric conductor boundary condition (PEC boundary condition) was placed in the y-direction. The performance of the unitcell was then gauged by illuminating the unitcell and then recording $|\text {S}_{11}|$.

**Figure 2:**
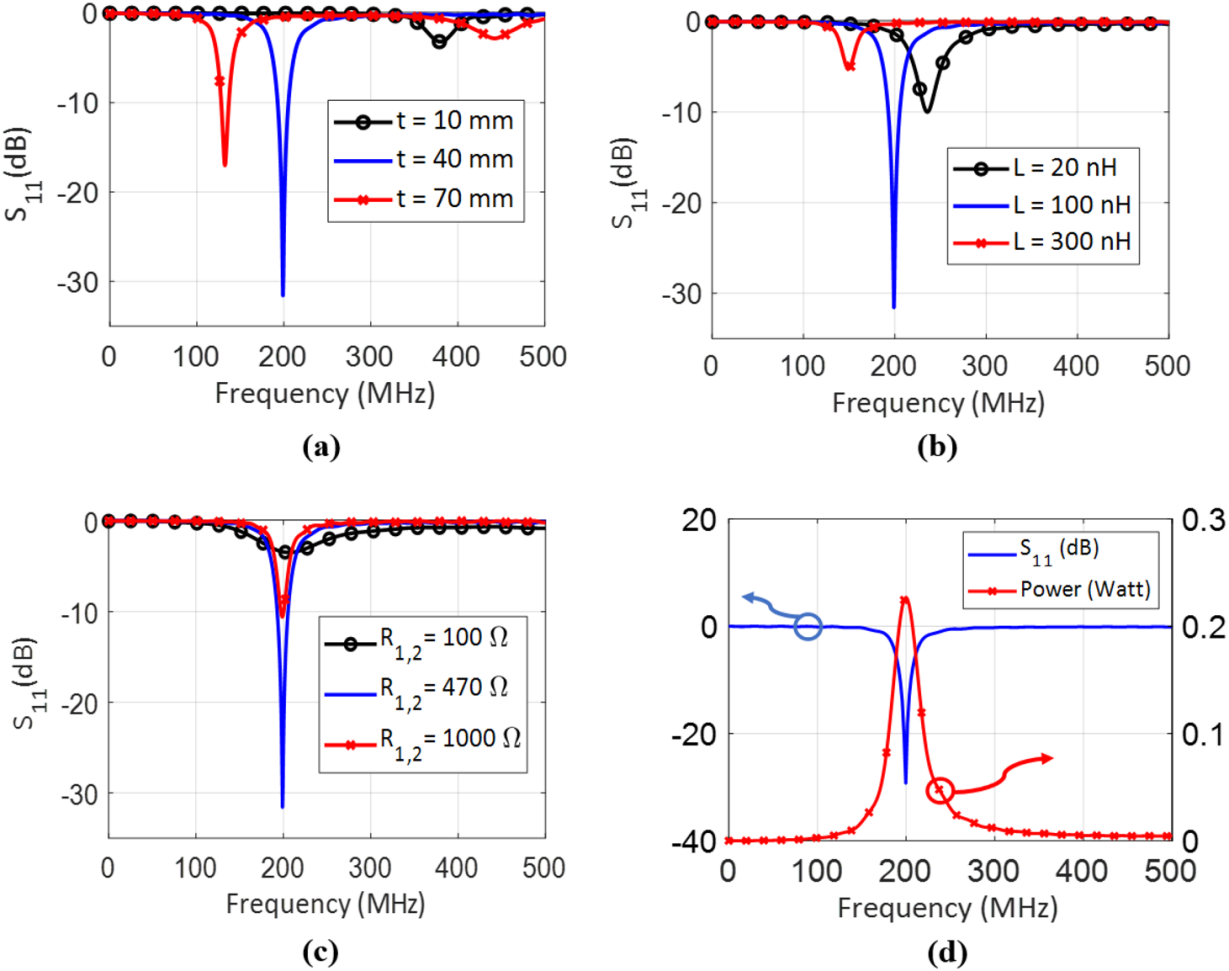
$|\text {S}_{11}|$ for different values of (**a**) $\textrm{t}$, (**b**) $\textrm{L}$, and (**c**) $\mathrm {R_1=R_2}$, where the non-variant parameters are set to $\mathrm {L=100}$nH, $\mathrm {R_1=R_2 = 470 \Omega }$, and $\mathrm {t=40}$ mm. (**d**) $|\text {S}_{11}|$ and power delivered to the terminal resistors of the optimized unitcell, where the incident power is 0.5 Watts.

The unitcell was optimized based on two key targets, minimum $|\text {S}_{11}|$ and maximum Q-factor. The optimized parameters of the unitcell are given in Table 1; this unitcell has a footprint of 10.5 mm $\times$ 10.5 mm and a thickness of 4.0 cm, which allows for increased inductance due to the long vias. Figure 2 shows the response of the unitcell, where three controlling parameters were investigated: the substrate thickness $\textrm{t}$, the inductors’ inductance values $\mathrm {L_1 = L_2 = L_3 = L_4 = L}$, and the termination resistance values $\mathrm {R_1 = R_2}$ (see Fig. 1).

In Fig. 2a, due to the fact that the vias will add inductance to the structure, the longer the vias are (i.e. higher $\textrm{t}$), the lower the resonance frequency would be. Figure 2b shows that the resonance frequency can be adjusted by varying the inductance of the lumped inductors. Figure 2c illustrates that the minimum of $|{\text {S}}_{11}|$ occurs for the value of $R_{1,2}$ = 470$\Omega$ at the resonance frequency of the unitcell, which indicates perfect impedance matching between the free space and the cell.

Figure 2a–c shows that the impedance matching is affected by the vias’ length ($\textrm{t}$), the inductors’ inductance $\textrm{L}$, and the termination resistors $\mathrm {R_1 = R_2}$, respectively. We further observe that for $\mathrm {t=40}$ mm, $\mathrm {L=100}$ nH, and $\textrm{R}_1=\textrm{R}_2 = 470~\Omega$, $|\text {S}_{11}|$ has a minimum value of lower than $-30$ dB, corresponding to a very strong impedance matching at 200 MHz, and a Q-factor of 16. This strong absorbance (matching) will not happen for other values of the parameters under study in Fig. 2.

Figure 2d shows the response of the optimized unitcell with the parameters given in Table 1. It can be seen that 0.46 Watts (92%) out of 0.5 Watts incident power is delivered to the termination resistors (0.23 Watt in each resistor), and 0.04 Watts (8%) is lost due to dielectric and ohmic loss.

## LFEM imaging system design

**Figure 3:**
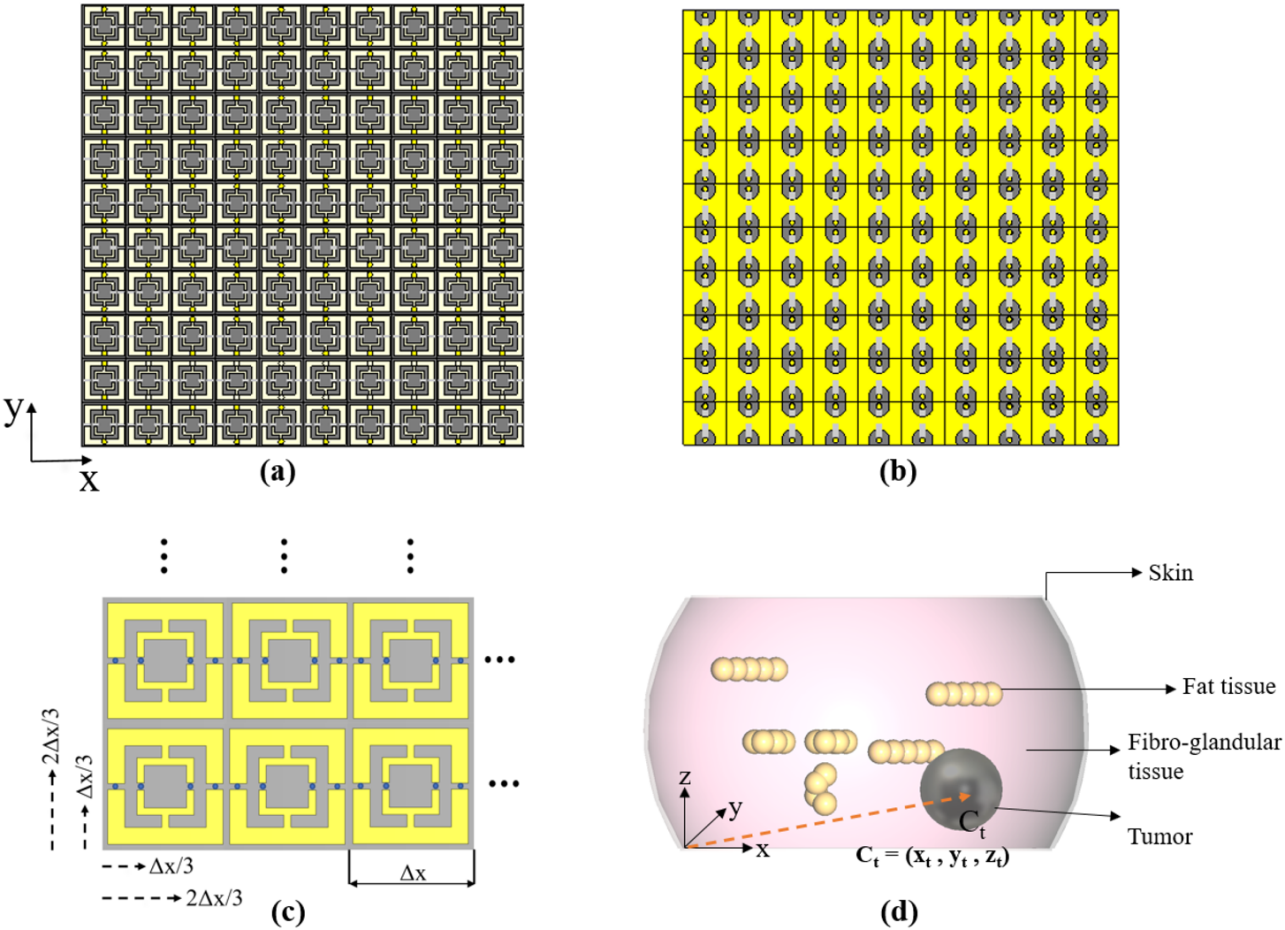
Metasurface array: (**a**) top view and (**b**) bottom view, showing the two termination resistors (**c**) scanning technique for increasing the pixels of the impression, where $\Delta$x is the length of the square unitcell. The arrows show the directions of the displacements. (**d**) Breast model, including skin, fat tissues, fibro-glandular tissues, and a tumor positioned at $C_t$.

A metasurface was formed as a 10 $\times$ 10 array of the unitcell described above. Figure 3a,b shows the top and bottom views of the metasurface. Each unitcell represents a pixel, the value of which will be assigned by measuring the power dissipation at the corresponding termination resistors. Owing to the symmetry in the unitcell, the received power will be equally distributed between the resistors. Thus, by measuring the dissipated power in one of the two termination resistors of a unitcell, and repeating the same measurement for all of them, we will end up with a two-dimensional power map, which can be thought of as an impression-image. In other words, the position ($\text {x}_\text {n}$, $\text {y}_\text {n}$) of each unitcell and its termination power dissipation is reserved for the impression-image construction, which reflects the power absorbed by the unitcells (pixels).

We have leveraged the technique proposed in ^22^ to record a 30 $\times$ 30 impression instead of a 10 $\times$ 10 impression. Considering that the length of the unitcell is $\Delta$x, in addition to the reference position, we recorded the impressions by shifting the whole metasurface with the value of $\Delta$x/3 and 2$\Delta$x/3 in both x and y directions. By doing so, nine different combinations of metasurface positions will provide us with nine pixels for each unitcell. Thus, using the whole array, with the same metasurface, an impression with 900 pixels, rather than 100 pixels, will be created (see Fig. 3c).

In our previous works^31–33^, it was shown that in the near field, the resolution can dramatically exceed the Abbe diffraction limit. If the source of radiation is placed very close to the breast, the electromagnetic field that impinges upon the breast contains all polarizations. Therefore, under such excitation, and as opposed to a plane wave excitation where the impinging field is polarized in one direction, the interaction between the impinging fields and the breast constituents provides a scattered or secondary field that has higher information content.

By recalling the spring and mass model of a molecule interacting with EM waves, due to the formation of dipole moments inside the sample, molecular polarization will take place. Depending on the polarization of the incident field, molecules inside the breast will be affected differently. Thus, by having different polarizations generated by the source, the molecules of healthy and cancerous tissues will be excited, thus generating independent information leading to unique impressions on the metasurface. An electrically small dipole with a length of $\lambda$/10 and a diameter of $\lambda$/1000, placed very close to the breast, was used as the radiation source.

In the numerical simulations, various types of breast models were considered to cover human female breast diversity. The breast mainly consists of fibro-glandular and fat tissues^34^. The density of the breast model can be categorized based on the density of the fibro-glandular tissues. Figure 3d shows the breast model used in the CST simulation, consisting of skin, fat tissue, fibro-glandular tissue, and tumor. Four different categories of the breast model were considered: (1) extremely dense breast (more than 75% fibro-glandular tissue), (2) heterogeneously dense (50–75% fibro-glandular tissue), (3) fibro-glandular scattered areas (25–50% fibro-glandular tissue), and (4) entirely fatty (less than 25%)^34,35^. The percentage of the fat and fibroglandular tissues can vary widely amongst women. However, the mean composition of the fibroglandular tissues, including skin, varies from 13.7 to 25.6% with the overall mean of 19.3%^36^. This means that most of the women in the statistical population are classified under entirely fatty category, which makes MWD process less challenging. In fact, the contrast between the fibroglandular and tumorous tissues is not as significant as the contrast between the fat and tumorous tissues. Thus, the examination of extremely and heterogeneously dense breasts is more difficult than that of fibroglandular-scattered and entirely fatty breasts^37^.

**Table 2:** Various locations of the tumor.

Descriptive location	Coordinate of the tumor’s center
Upper-left corner ($\text {C}_{\text {t1}}$)	(70 mm, 15 mm, 15 mm)
Upper-right corner ($\text {C}_{\text {t2}}$)	(20 mm, 15 mm, 15 mm)
Lower-left corner ($\text {C}_{\text {t3}}$)	(70 mm, 35 mm, 15 mm)
Lower-right corner ($\text {C}_{\text {t4}}$)	(20 mm, 35 mm, 15 mm)

For the purpose of validating our concept, the breast is considered to be a hemisphere with a radius of 50 mm, covered with a layer of skin with a thickness of 2 mm. The internal composition of the breast consists of fibro-glandular and fat tissues. At the operating frequency of 200 MHz, the relative permittivity and electrical conductivity for the fibro-glandular tissue are close to 64 and 0.8 S/m, whereas for fat tissue, they are close to 5.6 and 0.03 S/m^38^.The center $\text {C}_\text {t}$ of the spherical tumor, modeled as a perfect conductor (PEC), has been placed at ($\text {x}_\text {t}$, $\text {y}_\text {t}$, $\text {z}_\text {t}$) with reference to the origin of the cartesian coordinate system shown in Fig. 3d. This is a good preliminary electromagnetic model to guarantee sufficient contrast between the tumor and healthy tissues^39^. In Table 2, the locations of the tumor and their corresponding coordinates are listed. These locations were used to produce the dataset for the classification exercise.

## Numerical simulation, results, and discussion

**Figure 4:**
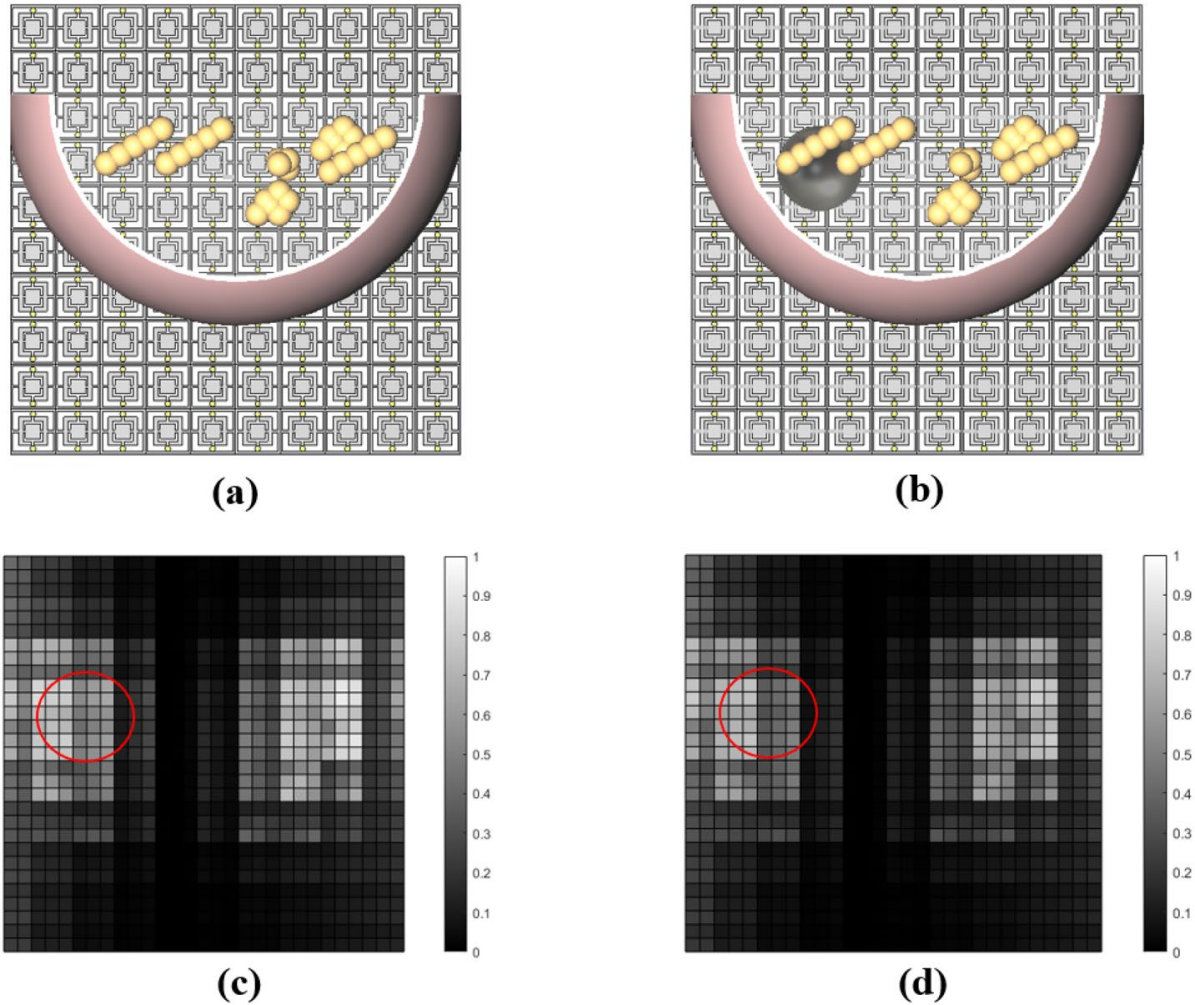
(**a**) Simulation model of the healthy breast (**b**) simulation model of the tumorous breast (**c**) recorded impression of the healthy breast (**d**) recorded impression of the tumorous breast, where the red circles indicate the border of the tumor with a radius of 10 mm and location of $\mathrm {C_{t_1}}$ (see Table 2).

Figure 4 illustrates the impression of a healthy breast model and that of a breast with a 10 mm tumor placed at the upper-left corner ($\mathrm {C_{t_1}}$, see Table 2) of the breast model. Considering Fig. 4, we can see that the differences between the two impressions seem to be negligible to the naked eye. To enhance the resolution, and consequently the potential to increase the differentiation in the impression for the breast with and without the tumor, we subtract each impression of a tumorous breast from the impression of the same breast but without the tumor.

**Figure 5:**
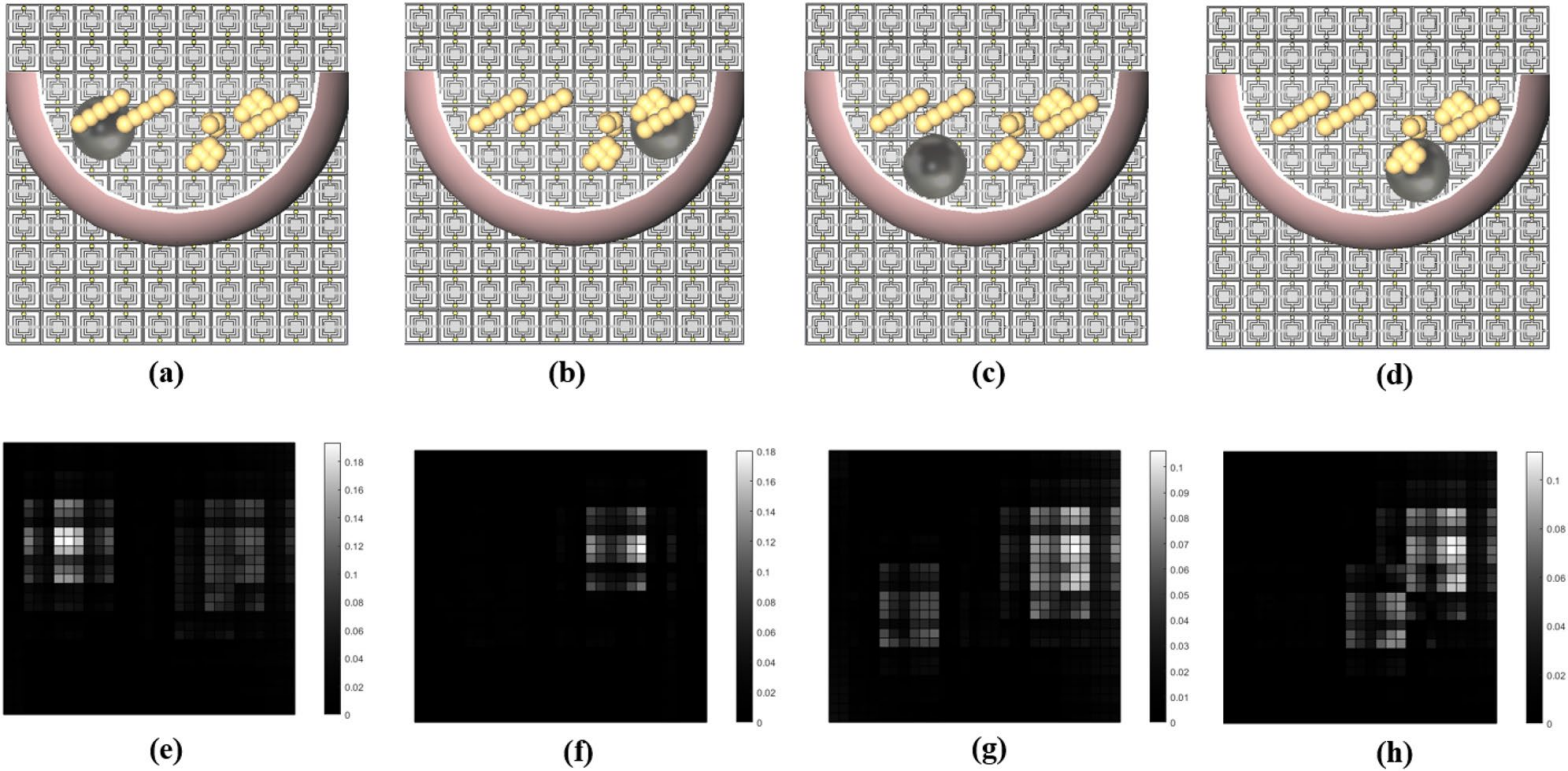
Model of the tumorous breast with a 10 mm tumor placed in: (**a**) upper-left corner, (**b**) upper-right corner, (**c**) lower-left corner, and (**d**) lower-right corner with the corresponding impressions shown in (**e**–**h**), respectively.

In the next step, we add a tumor sample with different dimensions in various locations. At first, a spherical tumor sample made of PEC with a radius of 10 mm is placed at four different locations: the upper-left corner ($\mathrm {C_{t_1}}$), the upper-right corner ($\mathrm {C_{t_2}}$), the lower-left corner ($\mathrm {C_{t_3}}$), and the lower-right corner ($\mathrm {C_{t_4}}$) of the breast (see Table 2 and Fig. 5a–d). We have selected these locations to show the quality of the impressions and their ability to detect the locations of the tumors and to better observe subtle dissimilitudes between different impressions.

**Figure 6:**
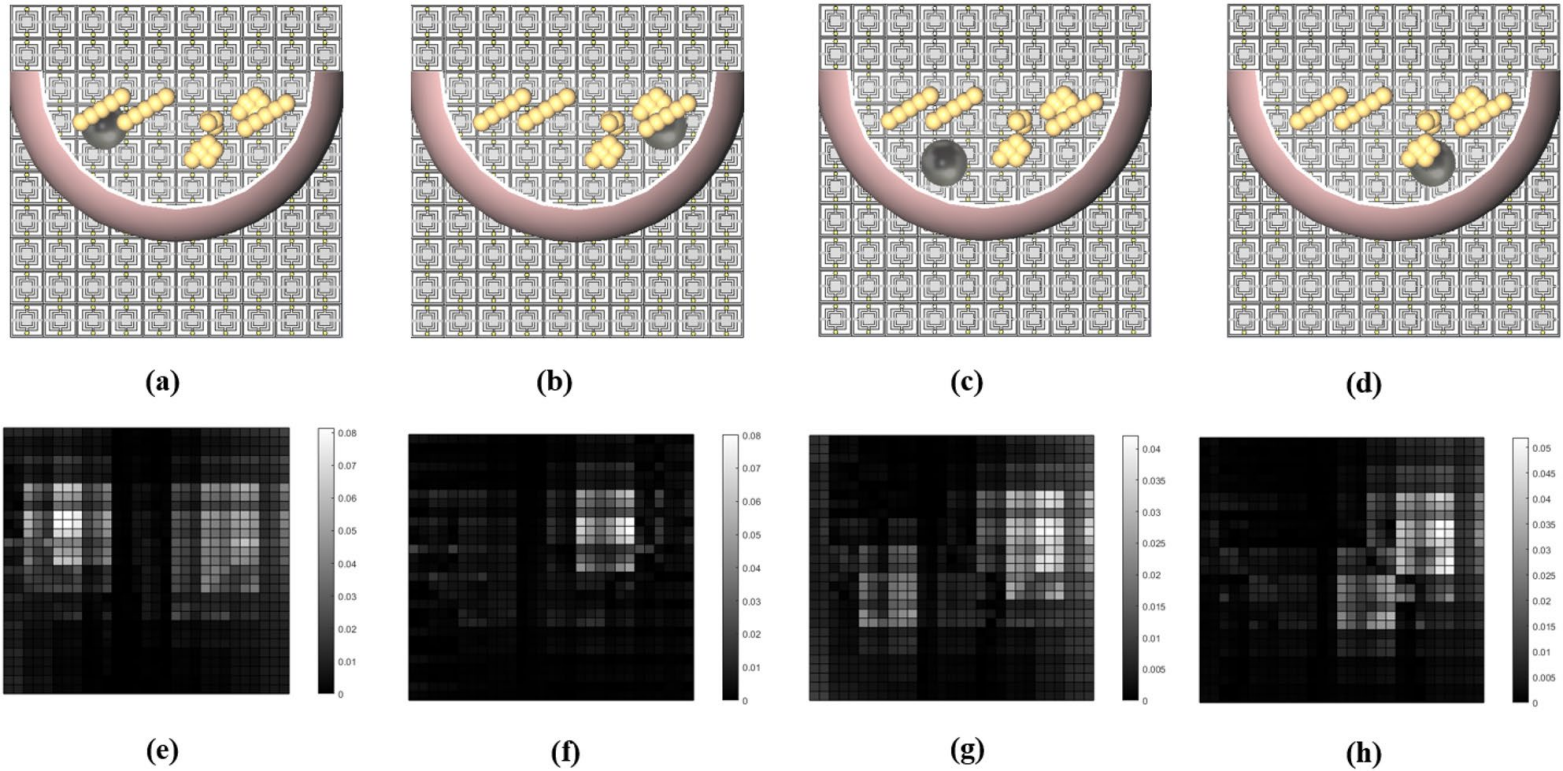
Model of the tumorous breast with a 7.5 mm tumor placed at: (**a**) upper-left corner, (**b**) upper-right corner, (**c**) lower-left corner, and (**d**) lower-right corner with the corresponding impressions shown in (**e**–**h**), respectively.

**Figure 7:**
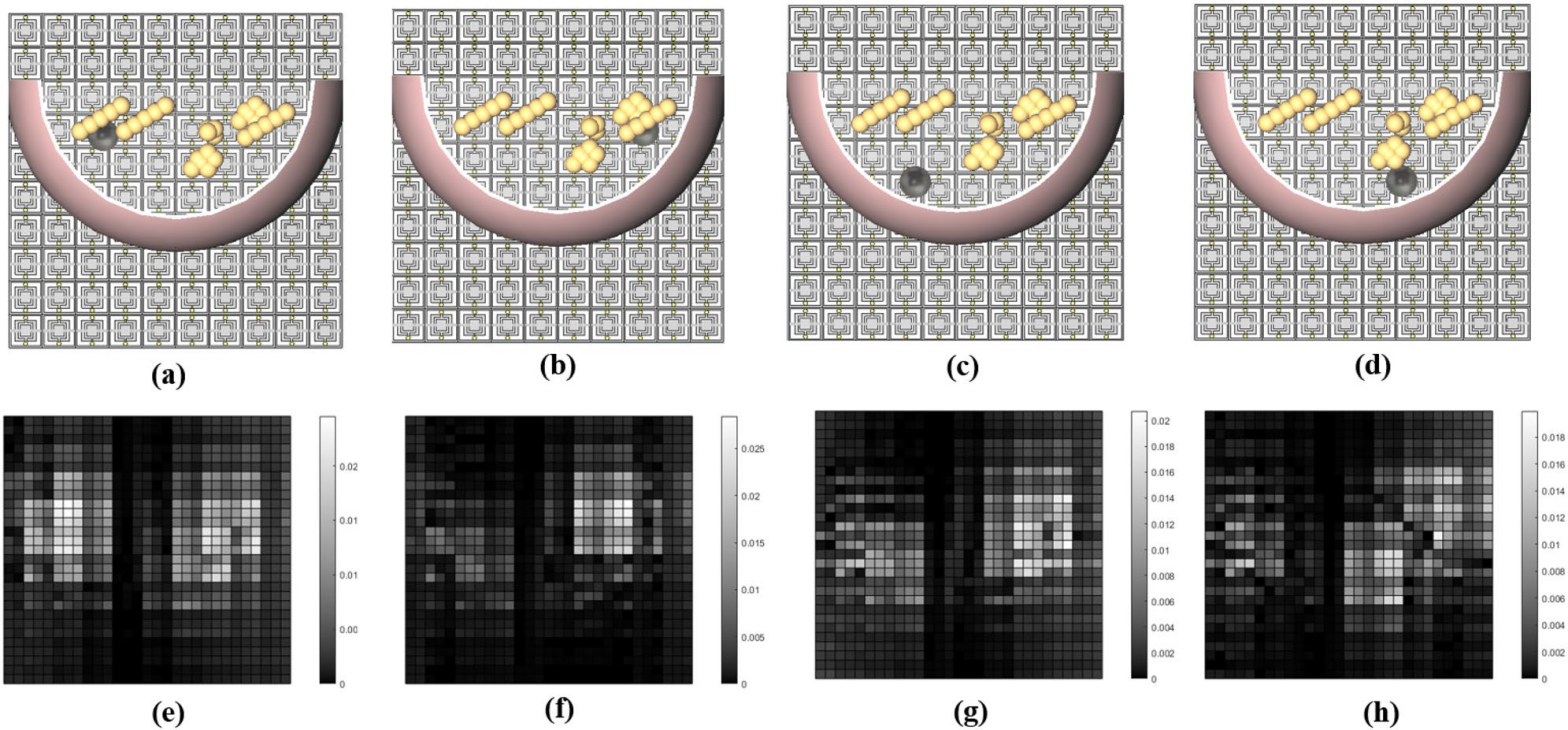
Model of the tumorous breast with a 5 mm tumor placed at: (**a**) upper-left corner, (**b**) upper-right corner, (**c**) lower-left corner, and (**d**) lower-right corner with the corresponding impressions shown in (**e**–**h**), respectively.

The effectiveness of the subtraction technique presented in this work is based on the assumption that the left and right breasts are identical for the vast majority of women. Statistically, the magnitude of relative BV (breast volume) asymmetry between the two breasts has a median of 2.71%, and the magnitude of relative DV (dense volume) asymmetry has a median of 3.28%^40^. By applying the subtraction technique mentioned above, we can observe in Fig. 5e–h that the most pronounced differences were around the tumor’s location. In Fig. 5e,f,h, we observe that a strong correlation exists between the impressions and the locations of the tumors. However, in Fig. 5g, the lightest pixels are somewhere different from the tumor’s location, although we can see dimmer differences around the location of the tumor. Next, we analyze the results of the simulations for four different locations of a tumor with a radius of 7.5 mm. The tumor was placed in one of the four positions given in Table 2 ($\mathrm {C_{t_1}}$, $\mathrm {C_{t_2}}$, $\mathrm {C_{t_3}}$, and $\mathrm {C_{t_4}}$) as depicted in Fig. 6a–d). Figure 6e–h show the impressions of the above-mentioned tumorous breast models after subtracting from the impression of the healthy breast. We observe that the most pronounced difference is close to the locations of the tumors. We also observe that the impressions cannot show the location of the tumors as accurately as those shown in Fig. 5e–h. The reason is due to the smaller size of the tumors, which makes them more challenging to be recognized from the corresponding impressions. Another noteworthy point here is that the difference in magnitude has been reduced, in the sense that the maximum contrast of the impressions has decreased from 0.18 to 0.08 for 10 mm and 7.5 mm tumor sizes, respectively (see the color bars of Figs. 5e–h and 6e–h).

Finally, we investigate the results of the simulations for four different locations of a tumor with a radius of 5 mm. The tumor was placed in the same positions (see Fig. 7a–d). Figure 7e–h show the impressions of the corresponding tumorous breast models after applying the subtraction method.

If the contrast in the achieved impressions is more than a DV asymmetry of 3.28%, there should exist some anomalies in the female breast. From the simulations, a contrast of more than 3.28% for 7.5 mm and 10 mm tumor sizes, was obtained, which confirms the effectiveness of the proposed modality in identifying an anomaly with such dimensions. However, the impressions for a tumor with 5 mm radius do not show the same level of dissimilarity, thus making it more difficult to identify tumors having a radius less than 5 mm (see the color bars of Fig. 7e–h).

## Mammography system setup and phantom studies

**Figure 8:**
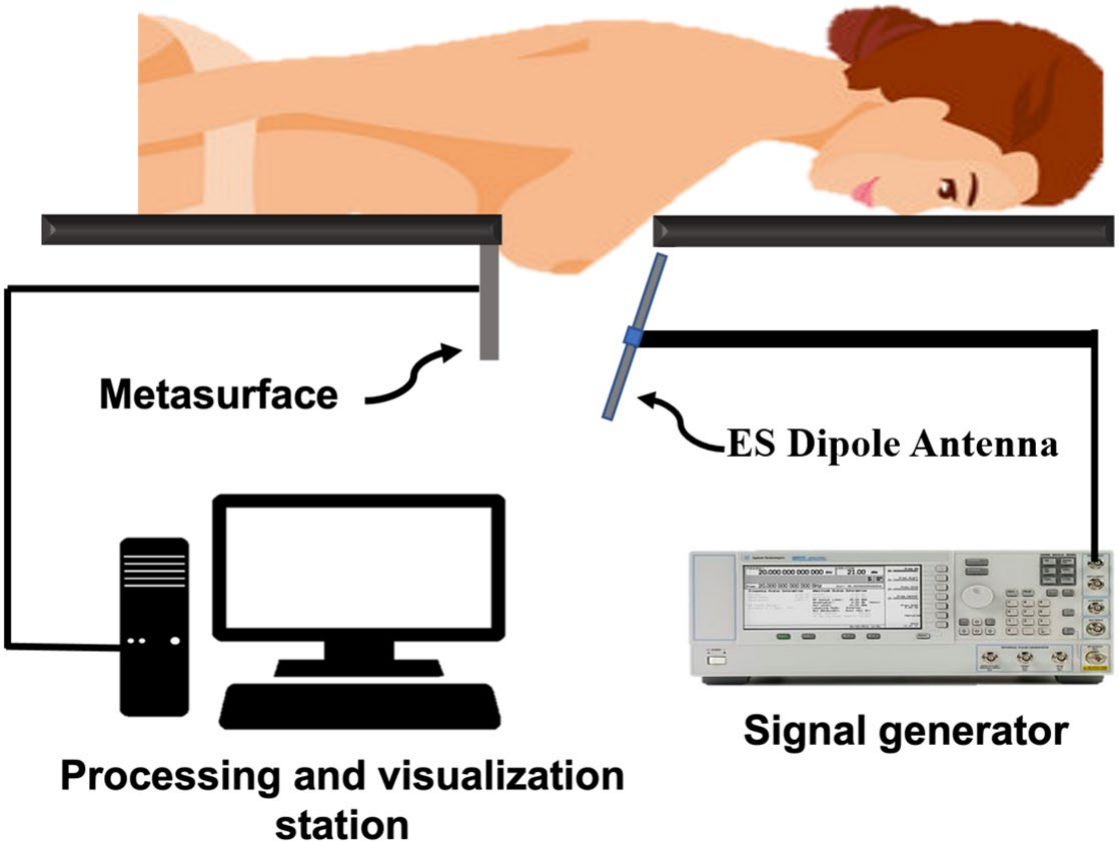
Mammography system setup.

Figure 8 shows the full mammography system setup in clinical investigations. The electrically small antenna (ESA) is fed by a CW signal at the desired frequency (200 MHz). Because ESAs are very inefficient radiators, only a small portion of the power from the signal generator will be radiated. The radiated power can be increased by employing a power amplifier. The radiated low-frequency electromagnetic waves will penetrate into the female breast, and after interaction with the female breast constituents, the power incident on the metasurface will be recorded using a spectrum analyzer or a power meter. By recording the power from each unitcell, an impression (or power map) will be obtained for the breast.

A critical component of our mammography system is the metasurface. To validate the concept, the metasurface was built using a 10 $\times$ 10 array of unitcells, where each unit cell is 1 cm $\times$ 1 cm. The metasurface should incorporate sufficient number of unitcells (pixels) to provide sufficient resolution to capture small anomalies inside the female breast. (For a higher resolution, smaller unitcells would be needed, which would require further miniaturization of the unitcell.) It is also possible that we use a smaller array size (i.e. 5 $\times$ 5) to reduce the complexity of the circuit, but then, we need to fully scan the lower side of the breast by moving the metasurface, which adds the mechanical complexity of the system.

Regarding the female breast positioning in real-world clinical setup, the upper and lower sides of the breast will be slightly pressed (see Fig. 3d). Thus, in the z coordinate, we will not encounter any challenge in adjusting the position. In the x and y directions, it is important that the left and right breasts are positioned identically with respect to their corresponding coordinate system reference. This can be possible by having the woman under test to lie in prone position (see Fig. 8).

**Figure 9:**
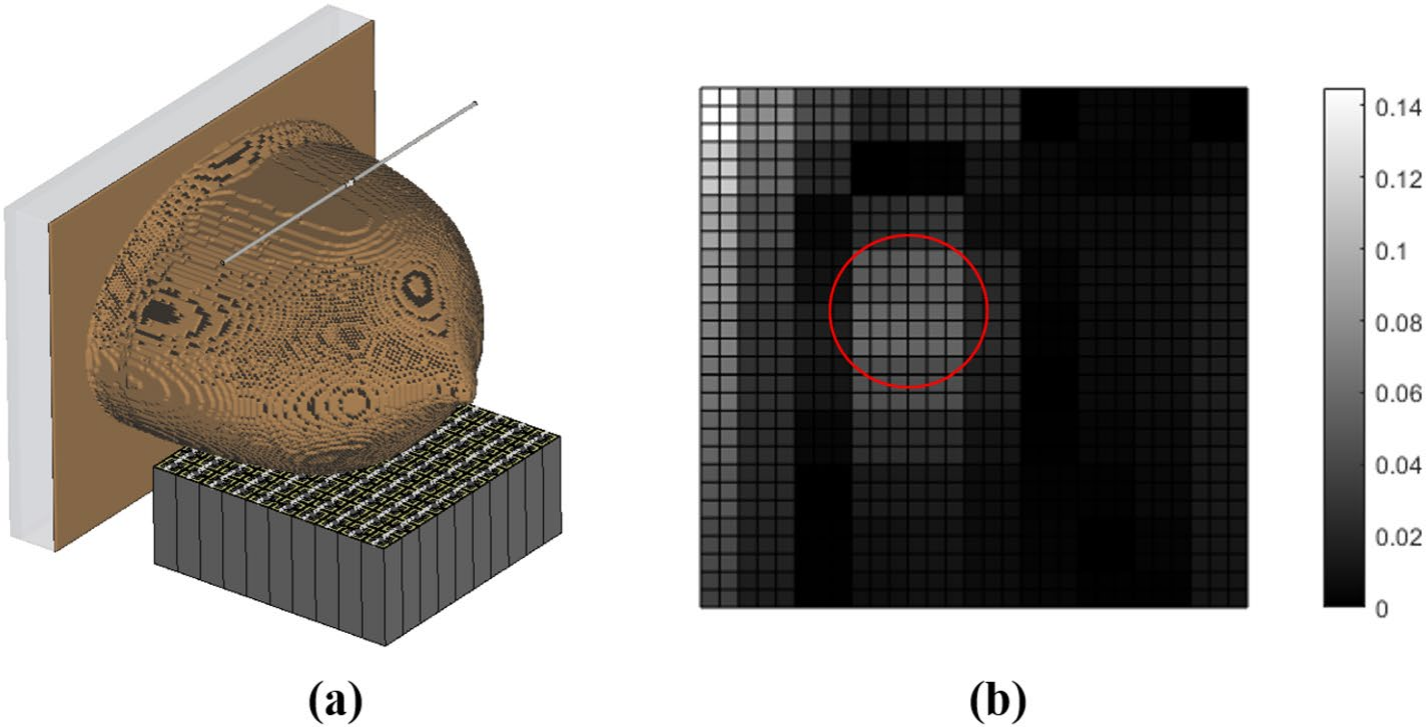
(**a**) The simulation setup of the ACR Class 2- Scattered Fibroglandular breast phantom (**b**) the obtained impression, where the red circle indicates the border of the tumor with a radius of 1.5 cm.

We emphasize that the proposed method is valid for any configuration of anomalies with any arbitrary shapes because it is intended for detection of anomalies within the breast rather than imaging of the entire breast. Furthermore, it is valid for any breast shape with various fibroglandular and fat tissues, as long as the minimum contrast (DV asymmetry) between the anomalies and healthy constituents exists. Here, we present a case study for a realistic numerical phantom model. This model is ACR Class 2- Scattered Fibroglandular breast phantom, the constituents of which have been translated from MRI sagittal slice into CST Microwave studio. It is consisting of 100 different fibroglandular voxel configurations, each of which represents specific electromagnetic characteristics. The valid frequency range of the numerical phantom is from 0 to 20 GHz^41,42^. The embedded tumor has a relative permittivity of 130 and an electrical conductivity of 2 S/m, which guarantees a low contrast of approximately 2:1 between the fibroglandular and cancerous tissues^18^. Figure 9 shows that by using this method, regardless of the breast model under investigation, we would achieve the sufficient contrast of 14% in the obtained impression, which is more than the DV asymmetry of 3.28%.

Considering the last section, where we investigated different scenarios of tumor sizes and locations, we conclude that by simply using the subtraction method, we cannot achieve highly reliable results in terms of a strong correlation between the tumor presence and the corresponding impression. Increasing the number of classes for different tumor sizes and locations will make distinguishing the impressions from each other more complex. Therefore, to provide better tumor detectability, in the next section, we apply a deep learning (DL) method to classify different impressions and separate them from each other.

**Figure 10:**
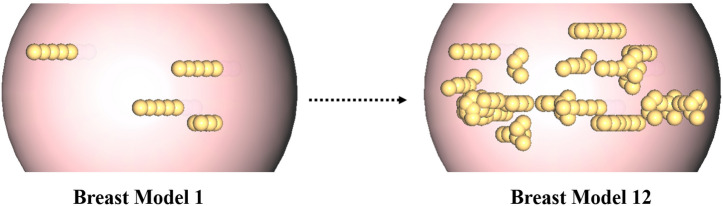
Increment of the fat tissues in the dataset breast models.

Since our objective is to detect the location and size of the tumors (not their shapes or numbers), for simplicity, we have chosen to do the simulations with spherical tumors and with different radii. The 12 breast models that we used in our dataset are different in their fat constituents. We started from an extremely dense breast model and gradually added fat tissues into the breast in order to make it fattier (see Fig. 10).

## Deep learning classifications

**Figure 11:**
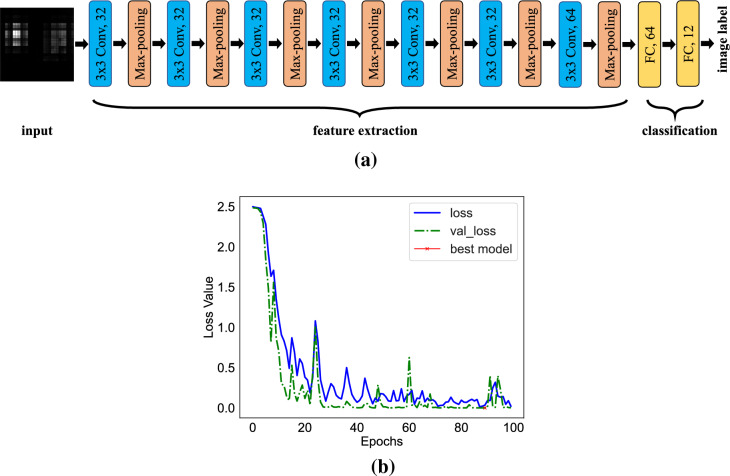
(**a**) Deep learning architecture composed of seven convolutional neural networks and two fully connected layers. (**b**) Graph of loss function’s values for training and validation sets over 100 epochs. The model with the least validation loss is marked with x and used for testing purposes.

We have developed a deep learning architecture to classify the impressions recorded from the simulations. Figure 11a illustrates the complete architecture of the DL classifier. The input impression is a grayscale of dimensions 30 $\times$ 30. Seven 3$\times$3 convolutional neural network blocks, each followed by a rectified linear unit (ReLU) and a 2 $\times$ 2 max pooling with stride 1 for downsampling compose the feature extraction part of the classifier. The last convolutional layer applies 64 filters, while other layers use 32 filters. No padding was used in this architecture. The features are then flattened and passed to fully connected layers with 64 nodes. A dropout with a rate of 0.5 is applied for regularization. We finally added the last fully connected layer with 12 nodes, which represent the number of classes, followed by a softmax activation function to predict image labels.

**Table 3:** Size and location of the tumors for different classes.

Class name	Size	Location
Class A	10 mm	$\mathrm {C_{t_1}}$
Class B	10 mm	$\mathrm {C_{t_2}}$
Class C	10 mm	$\mathrm {C_{t_3}}$
Class D	10 mm	$\mathrm {C_{t_4}}$
Class E	7.5 mm	$\mathrm {C_{t_1}}$
Class F	7.5 mm	$\mathrm {C_{t_2}}$
Class G	7.5 mm	$\mathrm {C_{t_3}}$
Class H	7.5 mm	$\mathrm {C_{t_4}}$
Class I	5 mm	$\mathrm {C_{t_1}}$
Class J	5 mm	$\mathrm {C_{t_2}}$
Class K	5 mm	$\mathrm {C_{t_3}}$
Class L	5 mm	$\mathrm {C_{t_4}}$

**Figure 12:**
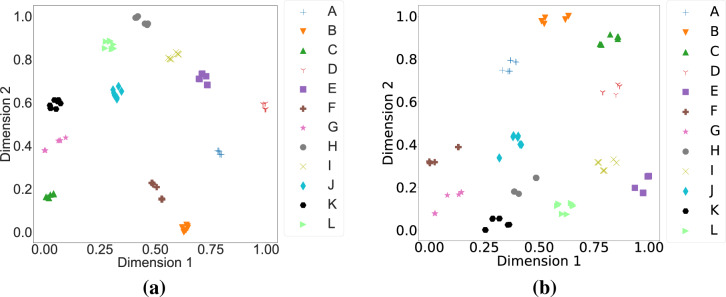
t-SNE visualization of the training data for twelve classes shown in Table 3. (**a**) Based on the features extracted from the deep learning architecture (**b**) Based on the HOG features.

We ran 144 simulation scenarios and obtained impressions from different models having different tumor sizes and locations. By changing the percentage of the fat tissue inside the breast model, we simulated different breast models ranging from extremely dense to fatty. In this work, we have used 12 different models, wherein tumor samples of three different dimensions were placed in four distinct locations inside the diverse breast models. Then, impressions obtained by each simulation scenario generated a dataset containing twelve classes, as shown in Table 3. The data was divided into training (66%), validation (17%), and test (17%). A total number of 100 epochs with a batch size of four was run to train the architecture. The model’s loss function on the training and validation cohorts are illustrated in Fig. 11b, where the best model was saved based on the validation loss and used for testing purposes. We used an Adam optimizer with a learning rate of $10^{-3}$ and a categorical cross entropy as the cost function. Sheer, zoom and rotational augmentations were used to enhance the model’s generalizability. We used a t-SNE dimensionality reduction to visualize different classes in two dimensions only. Figure 12a shows the results of the training data. It is seen that all of the twelve classes are distinctly separated from each other, indicating that the model successfully captured the critical features in the images based on their classes. The obtained F1-score and confusion matrix additionally resulted in 100% accuracy for all twelve classes, thereby confirming that our introduced model successfully distinguishes between classes. We further employed a decision tree model to compare its accuracy with that of our deep learning model. For this purpose, a histogram of gradients (HOG) approach with 12 bins as orientations is employed to extract the images’ features. The accuracy achieved amounts to 75%, as opposed to the perfect accuracy acquired by the deep learning algorithm. The t-SNE visualization of the extracted features using HOG is illustrated in Fig. 12b. It is evident that the t-SNE visualization of features generated by the deep learning implementation demonstrates a remarkable level of proximity among features belonging to different classes. This proximity suggests that the deep CNN effectively encodes essential patterns and distinctive characteristics, enabling it to return similar features for nearly all classes. On the contrary, the t-SNE representation of features obtained from the HOG algorithm exhibits a noticeable difference. The points corresponding to a specific class are notably more dispersed. This dispersion indicates that the HOG-based features may not be as effective in capturing and preserving the inherent relationships between samples belonging to the same class.

## Conclusion

This work demonstrated a new technology for detecting the anomalies inside human female breast. The proposed detection system is mainly based on a metasurface, consisting of miniaturized unitcells, operating at 200 MHz. The combination of an electrically small radiating source and the metasurface was able to provide distinguishable impressions for various scenarios (different tumor sizes and locations). The captured impressions, provided by the hardware (source and detector antennas), then, were fed into a deep learning algorithm to classify them automatically instead of manually. The output of the software achieved 100% accuracy.

While this work demonstrated the validity of the concept proposed here, in a future work, we will develop a prototype to demonstrate the feasibility of our detection modality experimentally.

## Methods

In this work, the miniaturized unitcell was designed by adding inductance to the split ring resonator. Then, the optimization was performed in both CST Microwave Studio and HFSS. Later, the whole system was simulated in CST, and the dissipated power values, in each terminated resistor, were recorded. The recorded data was transferred and plotted in MATLAB by applying the subtraction technique. Then, 144 different simulations were run in CST to make the dataset. Finally, the dataset were fed into the neural network to achieve higher detection accuracy.

## Data Availability

The dataset is available upon correspondence with O.M.R. (oramahi@uwaterloo.ca).
